# Development and validation of LRP1B mutation-associated prognostic model for hepatocellular carcinoma

**DOI:** 10.1042/BSR20211053

**Published:** 2021-08-31

**Authors:** Jian Xu, Xiaomin Shen, Bo Zhang, Rui Su, Mingxuan Cui, Lihua Yan, Yu Cao

**Affiliations:** 1 Tianjin Second People’s Hospital, Tianjin 300192, China; Tianjin Institute of Hepatology, Tianjin 300192, China; 2 Department of Immunology, Tianjin Key Laboratory of Cellular and Molecular Immunology, School of Basic Medical Sciences, Tianjin Medical University, Tianjin, China; 3 School of Pharmacy, Tianjin Medical University, Tianjin, China

**Keywords:** hepatocellular carcinoma, immune cell infiltration ratio, immune checkpoint, LRP1B, prognostic model

## Abstract

**Purpose:** To develop a lipoprotein receptor-related protein 1B (*LRP1B*) gene mutation-based prognostic model for hepatocellular carcinoma (HCC) patients risk prediction. **Methods:** The LRP1B gene mutation rate was calculated from HCC patient samples. Meanwhile, differentially expressed genes according to LRP1B mutant were screened out for prognostic model establishment. Based on this innovative model, HCC patients were categorized into high- and low-risk groups. The immune status including immune cell infiltration ratio and checkpoints have been explored in two groups. The functions of LRP1B and risk factors in the model were verified using both *in vivo* and *in vitro* experiments. **Results:** It could be demonstrated that LRP1B was a potential negative predictor for HCC patients prognosis with high mutation frequency. The functions of LRP1B were verified with ELISA and Quantitative Real-time PCR method based on clinic-recruited HCC participants. Eleven genes displayed significant differences according to LRP1B status, which could better predict HCC patient prognosis. The functions of these genes were examined using HCC cell line HCCLM3, suggesting they played a pivotal role in determining HCC cell proliferation and apoptosis. From the immune cell infiltration ratio analysis, there was a significant difference in the infiltration degree of seven types of immune cells and two immune checkpoints between high- and low-risk HCC patients. **Conclusion:** The present study hypothesized a potential prognostic biomarker and developed a novel LRP1B mutation-associated prognostic model for HCC, which provided a systematic reference for future understanding of clinical research.

## Background

Hepatocellular carcinoma (HCC) is the fourth most abundant malignant tumor in the world as well as the second leading cause for the cancer-related human deaths, accounting for nearly 841000 new cases and 782000 deaths annually [1]. In the aspect of virus infection, the hepatitis B virus (HBV) and hepatitis C virus (HCV) are the critical causes for HCC development [2]. In addition to the viral hepatitis from HBV and HCV, there are still some non-viral risk factors that can induce the development of HCC [3]. Diabetes mellitus, alcohol abuse, cardiovascular disease, liver inflammation, obesity, dyslipidemia and non-alcoholic fatty liver disease (NAFLD) are some other major contributors to HCC development [4–6]. Although the prospects for better diagnosis and treatments of HCC have dramatically improved over the past decade, a comprehensive understanding of the pathogenesis of HCC remains a substantial impediment.

LRP1B represents lipoprotein receptor-related protein 1B gene, which has been initiated as a new tumor suppressor candidate gene [7]. It may inhibit tumor cell infiltration and metastasis by antagonizing the extracellular UPA system to hydrolyze proteins, degrading EMC, and preventing cell movement [8]. Studies have found that changes in the gene cause nearly 40% of the non-small cell lung cancer cell line [9]. At the same time, it was suggested that LRP1B was down-regulated in colon cancer tissues and suppressed the cell proliferation, migration and metastasis of colon cancer cells, which was closely associated with β-catenin/TCF signaling [10]. Taken together, several studies have demonstrated the suppressive roles of LRP1B in the cancer progression, implicating that restoring the function of LRP1B would be a promising strategy for the cancer treatment. However, the relation between LRP1B and HCC is still poorly understood.

Based on the fact of poor accuracy of prognosis, the HCC has brought great difficulty to clinical treatment. Since LRP1B has attracted more and more attention in the cancer research currently, a more comprehensive approach to build an LRP1B mutation-associated prognostic model for HCC is required. To address these issues, in the present study, the mutation rate of LRP1B in HCC patients and differentially expressed genes have been investigated deeply by a combination of bioinformatics and machine learning method to build an innovative HCC prognostic model. Furthermore, the function of LRP1B in HCC development was examined in clinically recruited participants. Meanwhile, the crucial factors in prognostic model were verified in HCC cell line HCCLM3. Moreover, the 22 specific immune cells as well as 6 immune checkpoints in LRP1B mutated HCC have been also studied comprehensively. All of these promising outcomes enriched the precise prognosis of the disease, which provided a tremendous help for future HCC study.

## Materials and methods

### Data source

There were two data sources for the present study. The first one was from TCGA database. We downloaded the MAF file for mutation information of 365 HCC patients from the database: **The Cancer Genome Atlas** (TCGA, https://tcga-data.nci.nih.gov/tcga/), of which 357 patients had complete survival information, which was used for the following analysis. In addition, we also obtained 237 HCC patients with complete clinical as well as mRNA expression information from ICGC database (https://icgc.org/), numbered Liver Cancer-RIKEN, JP (LIRI-JP).

The second data source was from clinically recruited HCC patients as external experiments to verify the results. A total of 126 patients with HCC were randomly selected in Tianjin Second People’s Hospital from January 2018 to December 2020. Excluding 6 patients, 120 HCC patients were included in the experiments. The patients were categorized into Grade I, Grade II, Grade III, and Grade IV using the traditional WHO classification. There was no significant characteristics difference between groups (*P>*0.05). The details are shown in Supplementary Table S1.

All patients in the present study met the following criteria: age 40–80; signed informed consent; met the clinical diagnostic criteria for HCC; complete clinical data; fulfilled inclusion criteria as: (1) positive for HBV and/or HCV antigens; (2) ≥2 cm in diameter of the liver mass, and one of the two imaging examinations of CT and MRI showed that the liver mass had typical features of HCC. If the diameter of the liver mass was 1–2 cm, CT and MRI were required for approval; (3) serum α-fetoprotein (AFP) ≥ 400 μg/l lasted for 1 month or ≥200 μg/l lasted for 2 months, and the elevation of AFP caused by other causes could be excluded, including pregnancy, germline embryonal tumor, active liver disease or secondary liver cancer etc.

The exclusion criteria were as following: (1) presence of malignancy other than HCC; (2) presence of other chronic liver disease; (3) absence of consent of patient or family. The study met medical ethics standards and was approved by the hospital’s ethics board, with the informed consent of patients or their families for all treatment and testing. The second data source was performed for the sequential function experiments (quantitative real-time PCR and ELISA examination) .

### LRP1B concentration detection with ELISA method

LRP1B concentration determination adopted ELISA double-antibody sandwich method, the specific operation was strictly carried out in accordance with the kit instructions (Abcam company): the sample was extracted from peripheral blood of HCC patients. The results of the determination were repeated three times independently. The prognostic stage of HCC patients was determined according to LRP1B concentration, which was the forecast staging.

### Quantitative real-time PCR

The HCC tissues were snap-frozen and stored in liquid nitrogen. The total RNA was extracted using RNAiso Plus (Takara, Beijing, China). The real-time PCR was performed with BrightGreen 2× qPCR MasterMix-Low ROX (ABM, Vancouver, Canada). PCR conditions were set up as follows: one cycle at 95°C 5 min; and 40 cycles at 95°C for 15 s followed by 60°C for 40 s. The relative expression was processed with the 2^−ΔΔ*C*_t_^ method.

### LASSO Cox regression analysis

Based on the expression values of differentially expressed genes, the Cox regression analysis was developed on the HCC samples, while the genes that are significantly related to the prognosis of HCC were screened with a threshold of *P*<0.01. Then the glmnet package of R language was generated for LASSO Cox regression analysis in order to further select the genes associated with the prognosis of HCC [11]. The selected genes were established for risk score based on the following formula:

$$\text{Risk score}=\sum_{\text{i}=1}^{\text{n}}{\text{ Coef}}_{\text{i}}\cdot {\text{x}}_{\text{i}}$$

Then the patients were divided into high- and low-risk groups according to the median of the risk score.

### Differential gene analysis

The differentially expressed genes analysis was based on the limma function package [12] of the R language (version 3.5.2), with the difference factor greater than 1.5-times and FDR ≤ 0.05 as a criteria.

### Cellular experiments

The HCC cell line HCCLM3 was purchased from ATCC Co. For siRNA transfection, specific LRP1B, CDCA8 and centromere protein A (CENPA) siRNAs (50 nmol/l) (OriGene) and a negative control were utilized. Lipofectamine 2000 (Sigma–Aldrich, U.S.A.) was used for transfection. The cells from each group were collected and evaluated for the proliferation assay using CCK-8 method (Fisher, China). The absorbance was evaluated at 450 nm using the plate reader purchased from Thermo Fisher Scientific Co. Meanwhile, the cell apoptosis was measured using flow cytometry after Annexin V-FITC/PI double staining. All experiments were repeated three times independently.

### Calculation of immune cell infiltration ratio

The software CIBERSORT was utilized to calculate the relative proportion of 22 immune cells in HCC patients [13]. The CIBERSORT software could use the deconvolution algorithm with the preset 547 barcodes to characterize the composition of immune infiltrating cells according to the gene expression matrix.

### Statistical analysis

The multifactor Cox regression model was developed to analyze whether risk score could predict the survival of patients with HCC independently. The Wilcoxon’s signed rank sum test method was used to compare the differences of immune cell infiltration. The statistical analysis was established by R software, with version number v3.5.2.

## Results

### HCC patients with mutations in LRP1B had a worse prognosis

**Figure 1:**
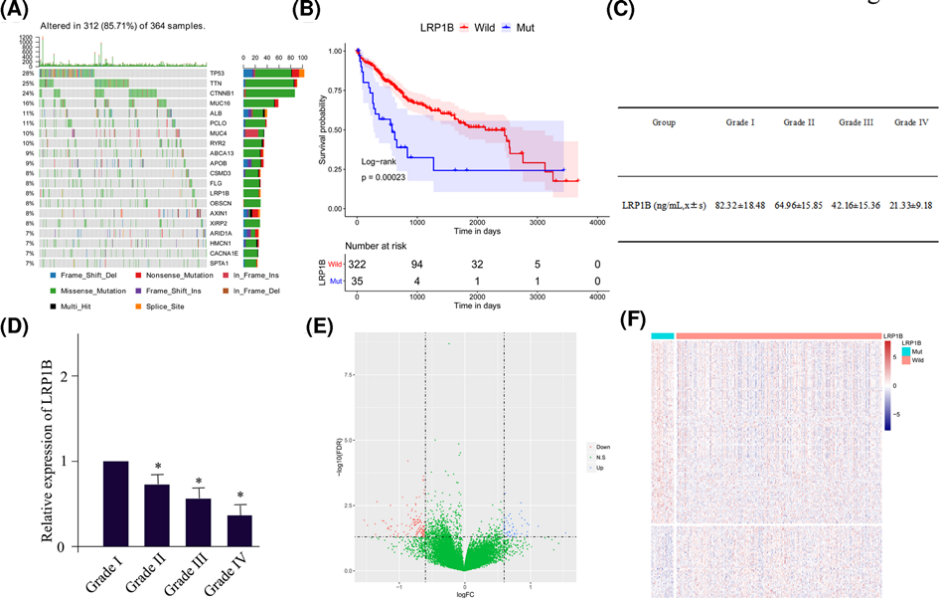
LRP1B mutation status is significantly related to prognosis (**A**) Waterfall plot of the top 20 genes with the highest liver cancer mutation rate in the TCGA sample. (**B**) Kaplan–Meier survival curve. The horizontal axis is time, the vertical axis is survival rate, respectively. Different colors represent different groups. The *P*-value is calculated based on the log-rank test. (**C**) ELISA for the LRP1B expression level with different Grades of HCC patients. (**D**) The quantitative real-time PCR measurement of LRP1B expression in HCC cancerous tissues. * indicates *P*<0.05 compared with HCC patients of Grade I. (**E**) The volcano map of differentially expressed genes. The horizontal axis is the differential expression fold (log2FC), while the vertical axis is −log10 (FDR), respectively. The blue dots indicate the up-regulated genes, and the red dots indicate the down-regulated genes. (**F**) Heat map of differentially expressed genes. The horizontal axis is the sample, the vertical axis is the different genes. At the same time, red indicates high gene expression, blue indicates low gene expression.

The mutation rate of *LRP1B* gene was high in TCGA patients with HCC, ranking at the 13^th^ place, reaching ∼8% (Figure 1A), and the overall survival (OS) of LRP1B mutant HCC patients was significantly lower than that of LRP1B wildtype HCC patients (Figure 1B). To confirm this in clinic, we examined the concentration of LRP1B using ELISA method. The expression level of LRP1B significantly declined as the increase in HCC Grade (Figure 1C). Since the circulating LRP1B was not specific to the liver function, we also investigated the LRP1B expression in HCC cancerous tissues using quantitative real-time PCR. Similar to ELISA outcomes, the activity of LRP1B was determined by the Grade of HCC (Figure 1D), which indicated the function of LRP1B as a negative prognosis factor for HCC patients. At the same time, we also analyzed the differentially expressed genes for LRP1B mutant patients compared with control individuals, concluding that 187 genes demonstrated specific expression manner in LRP1B mutant HCC patients. Among these, there were 134 up-regulated genes and 53 down-regulated genes as shown in Figure 1E,F.

### The risk model constructed by 11 genes could better predict the prognosis of HCC patients

**Figure 2:**
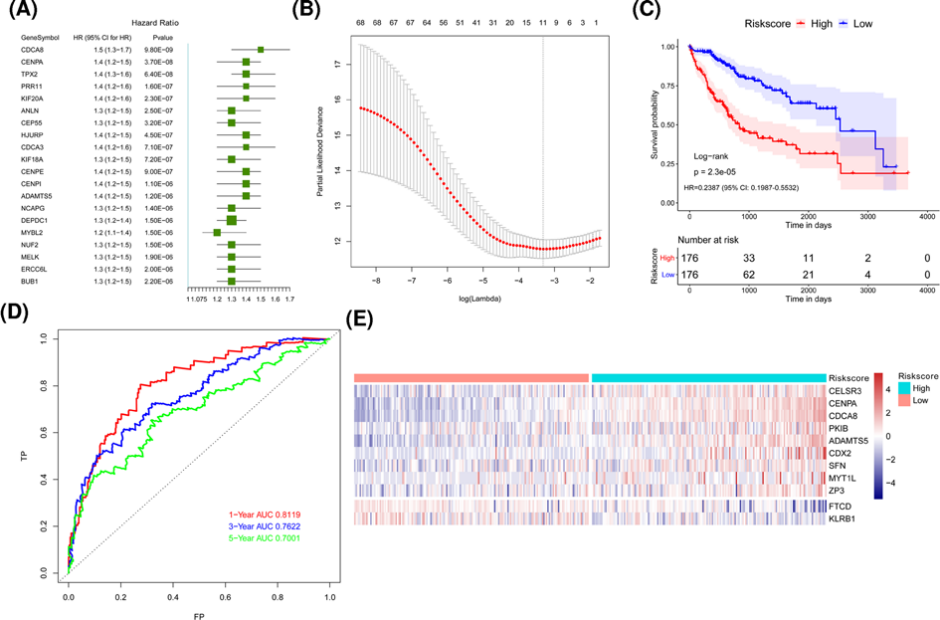
Construction of a prognostic model of HCC (**A**) Forest plot of the single factor analysis of the top 20 most significant genes related to the prognosis of HCC. HR is hazard ratio, 95% CI is 95% confidence interval. (**B**) The graph of determining the tuning parameter λ in the LASSO regression model. The horizontal axis is log (λ), and the vertical axis is the partial likelihood deviation value, respectively. The λ value corresponding to the smallest value is the best, which means that the best λ value after log is taken below the dotted line, and the number above is the number of variables. (**C**) Kaplan–Meier survival curve in the TCGA dataset, the horizontal axis is time, the vertical axis is survival rate. Different colors represent different groups. The *P*-value is based on the log-rank test. (**D**) The time-dependent roc curve, false positive on the horizontal axis and true positive on the vertical axis were used to estimate the area under the ROC curve (AUC). (**E**) The calorimetric maps of the mRNA expression of the 11 selected genes in the high- and low-risk score samples of TCGA dataset. The horizontal axis indicates the sample while the vertical axis indicates the gene. Meanwhile, the red represents the high expression, the blue represents the low expression, respectively. The categories of the samples are marked with different colors above the heat map.

Univariate Cox regression analysis was performed with 187 differentially expressed genes as continuous variables. At the same time, the hazard ratio (HR) of each gene was calculated. With *P*-value <0.01 as the threshold, 68 genes were finally selected out. Protective genes with HR value less than 1 were favorable for prognosis, while risk genes with HR value greater than 1 were unfavorable for prognosis. It was turned out that 3 of the 68 genes were protective genes, and the remaining 65 genes were risk genes. The forest map of the top 20 genes with the smallest *P*-value among these 68 genes was shown in Figure 2A. The optimal number of genes was 11 (Figure 2B, with the smallest λ value), 11 genes were cadherin EGF LAG seven-pass G-type receptor 3 (C*ELSR3*), killer cell lectin-like receptor B1 (*KLRB1*), *CENPA, CDCA8, PKIB, ADAMTS5, FTCD, CDX2, SFN, MYT1L* and *ZP3*, respectively.

The risk score model was established for predicting survival: risk score = (0.019437033*CELSR30) + (−0.138416034*KLRB1) + (0.070137596*CENPA) + (0.093717620*CDCA8) + (0.007794412*PKIB) + (0.166654021*ADAMTS5) + (−0.041895786*FTCD) + (0.054670700*CDX2) + (0.006845825*SFN) + (0.026858059*MYT1L) + (0.047343209*ZP3). We calculated the risk score for each patient and divided the TCGA dataset and the ICGC validation set into the high- and the low-risk groups according to the median of the risk score.

Survival analysis showed that in TCGA dataset and ICGC validation set, the high-risk HCC samples had the worse OS (Figure 2C). In addition, the AUC of 1-, 3- and 5-year survival time of TCGA dataset was 0.8119, 0.7622 and 0.7001, respectively (Figure 2D). The AUC of 1-, 3- and 5-year survival in ICGC validation set were 0.7182, 0.7297 and 0.7545, respectively, which indicated that the risk model could predict the prognosis of HCC patients effectively in both datasets. At the same time, we found that the expression of 11 genes was significantly different between high- and low-risk groups in TCGA and ICGC datasets (Figure 2E). In general, the risk score calculated from the risk model constructed by CELSR3, KLRB1, CENPA, CDCA8, PKIB, ADAMTS5, FTCD, CDX2, SFN, MYT1L and ZP3 could predict the prognosis of patients with HCC, with CENPA and CDCA8 as the two most significant factors.

### External experiments to verify the functions of factors in the risk score

**Figure 3:**
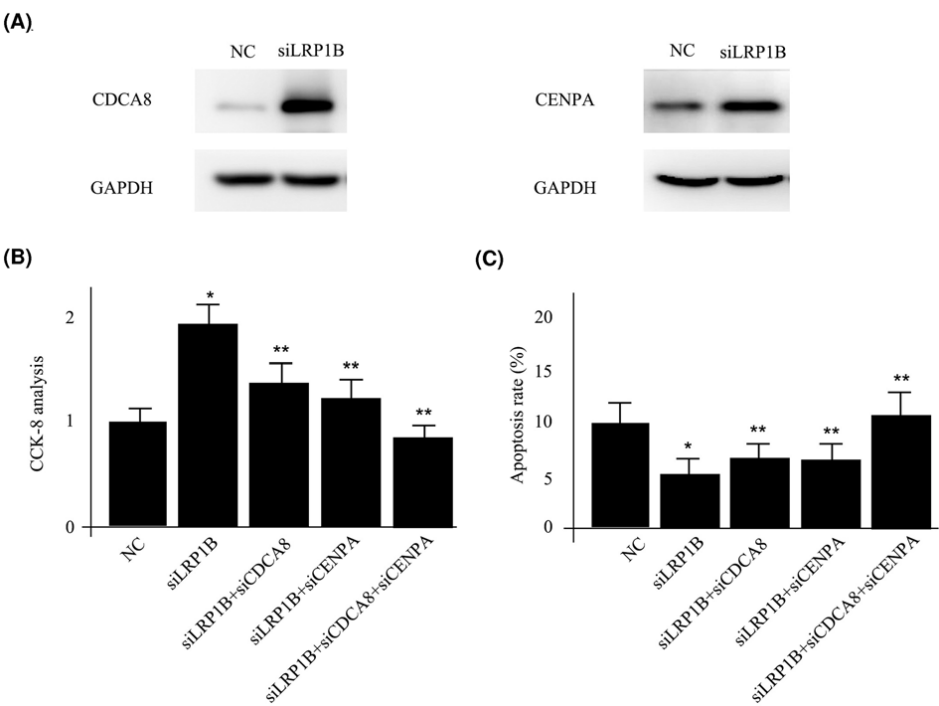
External experiments to verify the functions of factors in the risk score (**A**) Western blotting for CDCA8 and CENPA between negative control (NC) and siLRP1B transfections. (**B**) CCK-8 analysis for HCCLM3 cells with different treatments. (**C**) CCK-8 analysis for HCCLM3 cells with different treatments. * indicates *P*<0.05 compared with NC. ** indicates *P*<0.05 compared with siLRP1B transfection.

To verify the functions of factors from risk score in HCC formation, the HCC cell line HCCLM3 was utilized for endogenous investigation. As two most significant factors in the risk score model, the functions of CDCA8 and CENPA were elucidated deeply. As shown in Figure 3A, down-regulation of LRP1B by siLRP1B dramatically reduced the activities of CDCA8 and CENPA, which was consistent with the differential expression analysis. The efficiency and specificity of siLRP1B was examined as shown in Supplementary Figure S1. Moreover, reduced expression level of LRP1B could enhance HCC cell proliferation as well as decrease cell apoptosis, which was attenuated by co-transfection of CDCA8 or CENPA siRNA individually or a combination of two siRNAs (Figure 3B,C). All these together confirmed CDCA8 and CENPA as two potential targets for LRP1B mutant patients as well as for the risk score model.

### Risk score was an independent prognostic marker of HCC

**Figure 4:**
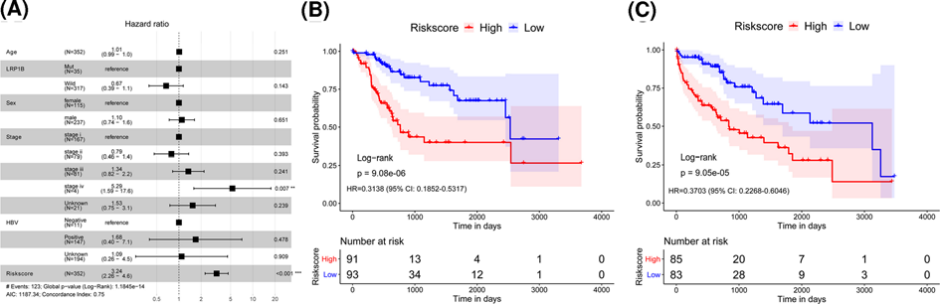
Risk score is an independent prognostic marker for HCC (**A**) Multivariate Cox regression analysis forest plot. Compared with reference samples, samples with HR greater than 1 have a higher risk of death, and samples with HR less than 1 have a lower risk of death. (**B**,**C**) Kaplan–Meier survival curve of HCC patients ≤60 and >60 years old.

We included age, sex, stage, LRP1B status, HBV index and risk score for the next investigation. The results are shown in Figure 4A. It could be found that risk score and age were significantly associated with OS, and the samples with high risk score had a higher risk of death and were unfavorable for prognosis (HR = 3.24, 95% confidence interval (95% CI): 2.26–4.6, *P*<0.001).

In order to further explore the prognostic value of risk score in HCC patients with different clinicopathological factors (including age and stage), we regrouped patients by age and stage, performing a Kaplan–Meier survival analysis. It could be demonstrated that the OS rate of the high-risk group was clearly lower than that of the low-risk group of the samples in different ages and stages (Figure 4B,C). These results confirmed that the risk score could be used as an independent indicator to predict the prognosis for HCC patients.

### Immunity status of HCC patients in high- and low-risk groups

**Figure 5:**
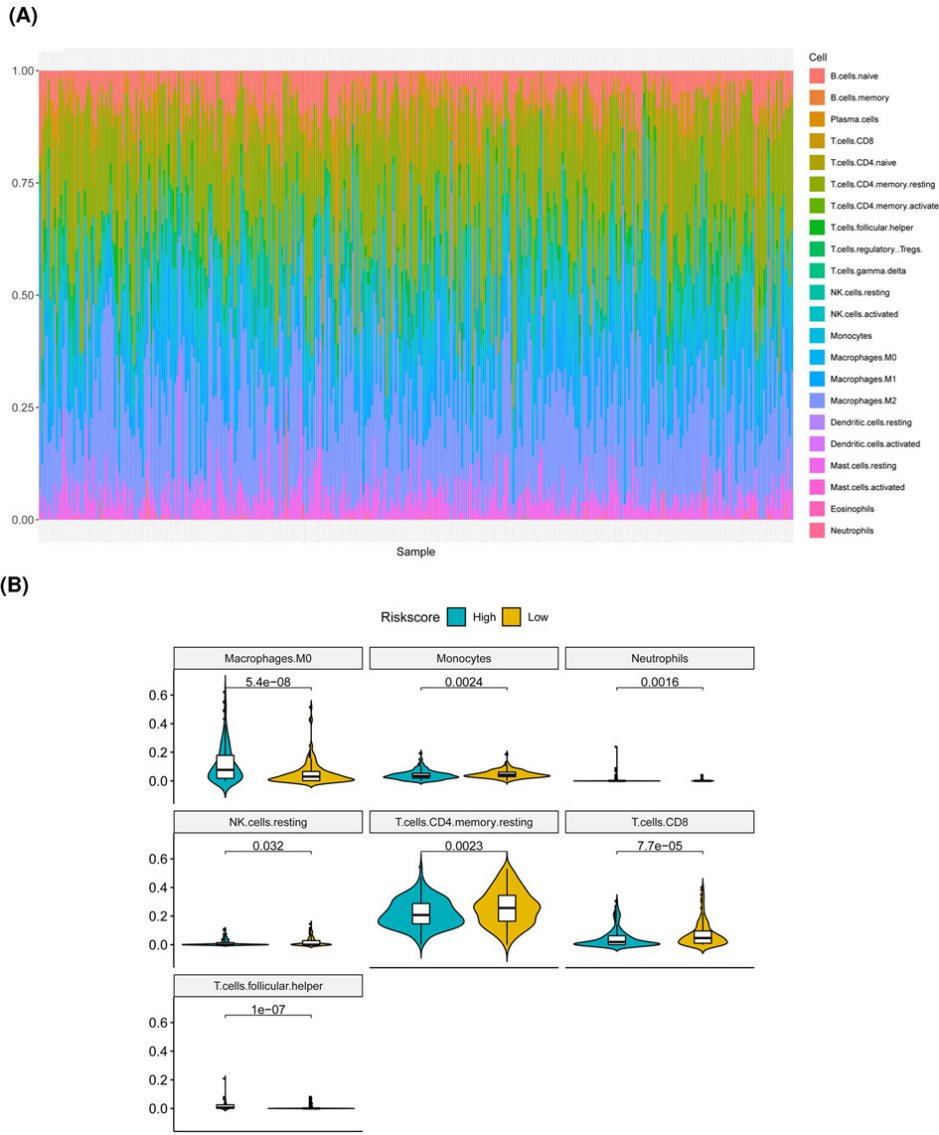
Immune infiltration of HCC patients in the high- and low-risk groups (**A**) The relative proportion of immune infiltrating cells in all patients. (**B**) The violin diagram of immune cells with significant difference in high- and low-risk groups. The horizontal axis represents high- and low-risk groups, the vertical axis represents relative infiltration ratio of immune cells, respectively. The *P*-value is calculated by Wilcoxon method.

We used the CIBERSORT method combined with LM22 feature matrix to estimate the difference of immune infiltration between 22 immune cells in high- and low-risk groups of patients with HCC. Figure 5A summarized the results of immune cell infiltration in 352 HCC patients from TCGA database. There were significant differences in the infiltration ratios of seven types of immune cells, such as macrophages (M0), between high- and low-risk groups (Figure 5B).

**Figure 6:**
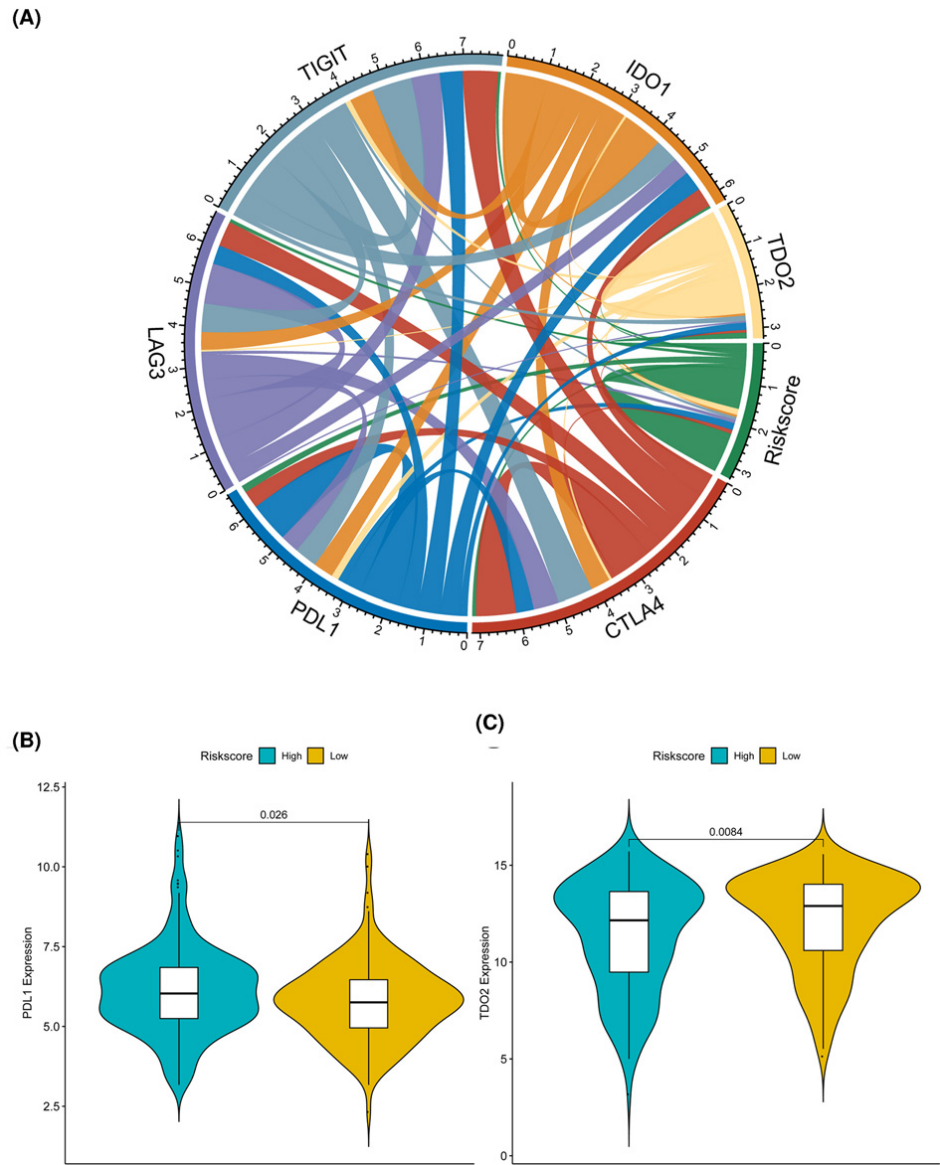
The relationship between several important immune checkpoints and risk score (**A**) Chord diagram of the correlation between the risk score and the expression of the six prominent immune checkpoints. The wider the line between them, the stronger the correlation between them. (**B**,**C**) The violin chart for PDL1, TDO2 and risk score. Different color indicates the high- and low-risk groups, while the vertical axis indicates the expression quantity. The *P*-value is calculated by Wilcoxon method.

Since the expression of immune checkpoints has become a biomarker of immunotherapy for HCC patients, we also analyzed the correlation between patient risk score and key immune checkpoints (CTLA4, PDL1, LAG3, TIGIT, IDO1, TDO2). It could be seen that the risk score was closely associated with the six checkpoints (Figure 6A). At the same time, two of the six immune checkpoints (PDL1, TDO2) had the significant differences in the high- and low-risk groups of HCC patients (Figure 6B,C).

## Discussion

HCC is the most common primary tumor of the liver and its mortality is third among all solid tumors, just behind carcinomas of the lung and the colon [14–17]. In this study, we established an innovative prognotic analysis based on LRP1B mutation. First of all, the mutation rate of *LRP1B* gene in HCC was shown to reach 8%, which was relatively high among all the mutations. Moreover, the majority of differentially expressed genes in LRP1B mutation HCC samples were risk genes, which were unfavorable for prognosis. These results were consistent with the function of LRP1B as a potential tumor suppressor. Previously, LRP1B has been studied in lung cancer as well as colon cancer [18]. A research by Ikari and colleagues suggested that LRP1B was one of most prominant somatic mutations in liver cancer metastasis, which initiated the key LRP1B function in HCC [19].

Based on the sequencing data of liver tumors from TCGA and next-generation sequencing (NGS) data of 657 liver tumors from Chinese clinical dataset, Wang and colleagues claimed that LRP1B contributed to 12.3% of HCC patients with mutated gene in the Chinese cohort [20], which is similar to the results here (shown in Figure 1A). Meanwhile, they also suggested that the LRP1B mutations were significantly associated with shorter OS (median, 20.9 vs 61.7 months; HR, 2.22; *P*=0.0012), as a poor prognostic factor in HCC. What is the internal connection between LRP1B and HCC? As a member of the low-density lipoprotein receptor family, LRP1B plays a tremendous function in maintaining lipid homeostasis. A study by Li and colleagues found that LRP1B knockdown caused the decrease in intracellular lipid content, down-regulated expressions of lipid synthesis-related enzymes as well as increased expressions of β-oxidation-related enzymes and enhanced activity of AMPK signaling [21]. These might offer a potential explanation for LRP1B roles in HCC development.

In addition to the prognosis model establishment for HCC, another feature of the present study was to explore multiple key associated genes. CELSR3 is the key signaling molecule in the wingless and INT-1/planar cell polarity (WNT/PCP) pathway [22]. It has been demonstrated as an organ-, inflammation- and cancer-specific transcriptional fingerprint of pancreatic and hepatic stellate cells [23]. Moreover, CELSR3 was suggested to be involved in the progression of cancer and can be used as a biomarker for the prognosis of HCC patients directly [24]. The *KLRB1* gene encodes for CD161 expressed by different subsets of leukocytes involved in the development of acute liver transplant rejection [25]. It has been promoted as a favorably prognostic gene by a pan-cancer resource and meta-analysis of expression signatures from 18000 human tumors with OS outcomes across 39 malignancies. CENPA stands for centromere protein A, which was initiated as a novel biomarker not only for HCC but also for lung cancer [26]. Altered expression of ADAMTS5 is associated with human carcinogenesis and tumor progression. ADAMTS5 has been approved to be expressed at differential levels in HCC cell lines, which also plays a role in suppression of HCC progression [27]. Beside these, the connections between the other genes and HCC remain unclear. All of these deserve further investigation.

Here, we also deeply investigated the immune cell infiltration in different groups of HCC patients distinguished by LRP1B mutation, where differences in the proportion of immune cell infiltration might be an intrinsic feature of individual differences receiving immunotherapy. In addition, the correlation between different types of immune cells was weak, which indicated that there was a large heterogeneity in the infiltration of different immune cells in tumor patients. Here, in the present study, seven types of immune cells, such as macrophages (M0) as well as two of the six immune checkpoints (PDL1, TDO2) had significant differences in the high- and low-risk groups of HCC patients, indicating that the poor prognosis of HCC patients with high risk might due to the immunosuppressive microenvironment. Immune checkpoints include stimulatory and inhibitory checkpoint molecules. The programmed cell death 1 ligand 1 (PD-L1) was suggested to function as an inhibitory checkpoint, which was identified to suppress anti-tumor immune responses in solid tumors [28]. Currently, several novel drugs targeting immune checkpoints have succeeded in cancer treatment. The combination of anti-PD-1/PD-L1 with anti-CTLA-4 antibodies is being evaluated in phase 1, 2 or 3 trials of HCC. At the same time, cancer epigenetic modulations of checkpoints also increase our understanding of potential therapeutic targets in related to the tumor microenvironment. Here, we explored the connection between these checkpoints to LRP1B mutant HCC, which may call for a great point for the future study.

To conclude, in the light of the fact that there remains no gold standard prognosis and no reliable disease-specific prediction for HCC, we established an innovative prognostic model for HCC based on LRP1B mutant patients. According to the model, several related genes and seven immune cells as well as two key immune checkpoints demonstrated significant difference in high- and low-risk groups. The functions of LRP1B and risk model have been verified in external experiments. Overall, we shed light on questions and challenges posed by the HCC, and we established a novel prediction target which could provide great help for future reference(s) of the disease.

## Supplementary Material

Supplementary Figure S1 and Table S1

## Data Availability

The datasets used or analyzed during the current study are available from the corresponding authors on reasonable request.

## Abbreviations

CELSR3: cadherin EGF LAG seven-pass G-type receptor 3
CENPA: centromere protein A
HBV: hepatitis B virus
HCC: hepatocellular carcinoma
HCV: hepatitis C virus
HR: hazard ratio
KLRB1: killer cell lectin-like receptor B1
LRP1B: lipoprotein receptor-related protein 1B
OS: overall survival
PD-L1: programmed cell death 1 ligand 1
TCGA: The Cancer Genome Atlas
