# Supplementary material for: Development and validation of LRP1B mutation-associated prognostic model for hepatocellular carcinoma
Source: Biosci Rep. 2021 Aug 31;41(9):BSR20211053. doi: 10.1042/BSR20211053 (PMC8415215; doi:10.1042/BSR20211053)
Supplement: Supplementary Figure S1 and Table S1 [file BSR-2021-1053_supp.pdf]

Supplementary Figure S1.

A

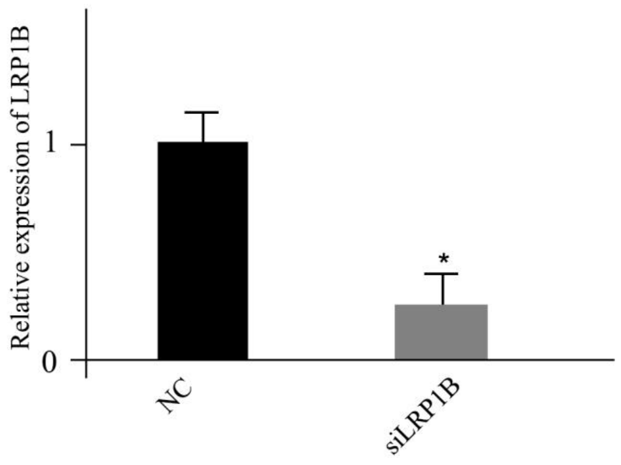

Supplementary Figure 1. The RT-PCR measurement for siLRP1B efficiency. NC： negative control. * indicates *P*<0.05 compared with NC.

Supplementary Table 1 clinicopathological characteristicsof 120 HCC participants

Characteristics	Groups	Patient	
		NO.	%
Sex	Male	76	63.3
	Female	44	36.7
Age at diagnosis	< 61(Median)	68	56.7
	>= 61(Median)	52	43.3
Pathological TNM stage	I	51	42.5
	II	36	30.0
	III	21	17.5
	IV	12	10.0
Adjacent hepatic tissue inflammation extent type	None	33	27.5
	Mild	42	35.0
	Severe	32	26.7
	Unknown	13	10.8
Person neoplasm cancer status	Tumor Free	76	43.4
	With Tumor	24	20.0
	Unknown	20	16.7
Vascular tumor cell type	None	52	43.3
	Micro	34	28.3
	Macro	18	15.0
	Unknown	16	13.3
